# Gestational age–dependent effects of intrauterine inflammation, delivery mode, and fetal growth on respiratory morbidity in preterm neonates

**DOI:** 10.3389/fped.2026.1823540

**Published:** 2026-08-04

**Authors:** Kyoko Yokoi, Osuke Iwata, Sachiko Iwata, Satoru Kobayashi, Kozue Kasukabe, Yasuhisa Nakamura, Kazuyuki Yamamoto, Takenori Katou, Shinji Saitoh, Haruo Goto

**Affiliations:** 1 Department of Pediatrics, Nagoya City University, West Medical Center, Nagoya, Japan; 2 Departiment of Pediatrics and Neonatology, Nagoya City University Graduate School of Medical Sciences, Nagoya, Japan

**Keywords:** gestational age, inflammation, large-for-gestational age, preterm neonate, respiratory morbidity

## Abstract

**Introduction:**

Respiratory morbidity in preterm neonates is influenced by both lung immaturity and antenatal environmental exposures. Because lung maturation continue throughout late gestation, the effects of these factors may vary according to gestational age. As most preterm neonates are born after 28 weeks, a better understanding of respiratory morbidty in this population is clinically important. We aimed to evaluate the gestational age–specific effects between intrauterine inflammation, other perinatal factors, and respiratory morbidity in neonates born between 28 and 36 weeks of gestation.

**Methods:**

We retrospectively analyzed the data of 912 neonates born between 28 and 36 weeks of gestation from March 2013 to December 2018 at Nagoya City University West Medical Center. Respiratory morbidity was defined as the need for invasive ventilation or continuous positive airway pressure for ≥24 h. Logistic regression analyses, including interaction terms with gestational age, were used to assess gestational age–specific associations.

**Results:**

Respiratory morbidity occurred in 275 neonates (30.2%) and was associated with lower gestational age, cesarean delivery, chorioamnionitis, funisitis, and low umbilical cord pH. At earlier gestational ages (33.4 weeks; 1 SD below the mean), intrauterine inflammation and higher birth-weight *Z*-score increased the risk of respiratory moribidity, whereas the adverse role of cesarean delivery was significant only at later gestational ages (36.5 weeks; 1 SD above the mean).

**Conclusion:**

The effects of intrauterine inflammation, fetal growth, and cesarean delivery on respiratory morbidity differed according to gestational age. These findings suggest that gestational age–specific assessment of perinatal factors may improve the prediction and management of respiratory morbidity in preterm neonates. However, because only gestational age was adjusted for, residual confounding by other factors cannot be excluded, and these findings should be interpreted with caution.

## Introduction

Approximately one-third to one-half of preterm infants experience cognitive or learning difficulties requiring support during school age or beyond (1), highlighting the gap between improved survival and suboptimal long-term outcomes. Inflammation, along with immaturity and hypoxia-ischemia, influences outcomes in preterm neonates through its association with morbidities such as periventricular leukomalacia, necrotizing enterocolitis, and chronic lung disease (2). The impact of inflammation on neonatal lungs has been extensively studied in extremely preterm neonates born before 28 weeks of gestation and in extremely low birth-weight neonates (3, 4). Preclinical studies have shown that the saccular stage of lung development is particularly vulnerable to inflammation (5). Neonates born between 28 and 36 weeks of gestation are predominantly in this developmental stage, during which alveolar and microvascular maturation remain ongoing (6). Experimental studies suggest that inflammatory exposure can disrupt ongoing alveolar and microvascular maturation, potentially affecting pulmonary function and respiratory adaptation after birth (7, 8). Consistent with these findings, intrauterine inflammation has been associated with an increased risk of bronchopulmonary dysplasia. However, its association with respiratory distress syndrome remains controversial, with previous studies reporting both higher and lower incidences of respiratory distress syndrome among exposed neonates (9–13). These inconsistent findings may reflect difference in clinical context, therapeutic interventions, and timing of exposure (12).

While substantial evidence is available regarding respiratory outcomes in extremely preterm neonates, relatively few studies have focused on more mature preterm neonates, likely due to their favorable survival outcomes. Both restricted and excessive fetal growth may influence lung maturation through alterations in the intrauterine hormonal environment (14), whereas cesarean delivery may affect postnatal respiratory adaptation by modifying the timing and physiological processes of birth (15). However, because 95% of preterm neonates are born after 28 weeks of gestation (16), improved prediction, diagnosis, and management of respiratory morbidity in very, moderate, and late preterm infants may substantially reduce healthcare burden. Because lung maturation continues throughout this period, the effects of various perinatal factors, including inflammation, on respiratory morbidity may vary according to gestational age. Accordingly, we aimed to evaluate the gestational age–specific effects of inflammation and other perinatal factors on respiratory morbidity in preterm neonates born after 28 weeks of gestation.

## Methods

This retrospective observational study was approved by the Ethics Committee of Nagoya City University West Medical Center, Nagoya, Japan (21-04-393-45). The review board waived the requirement for informed parental consent because anonymized data collected during routine clinical practice were used. All procedures complied with relevant guidelines and regulations.

### Study population

**Figure 1:**
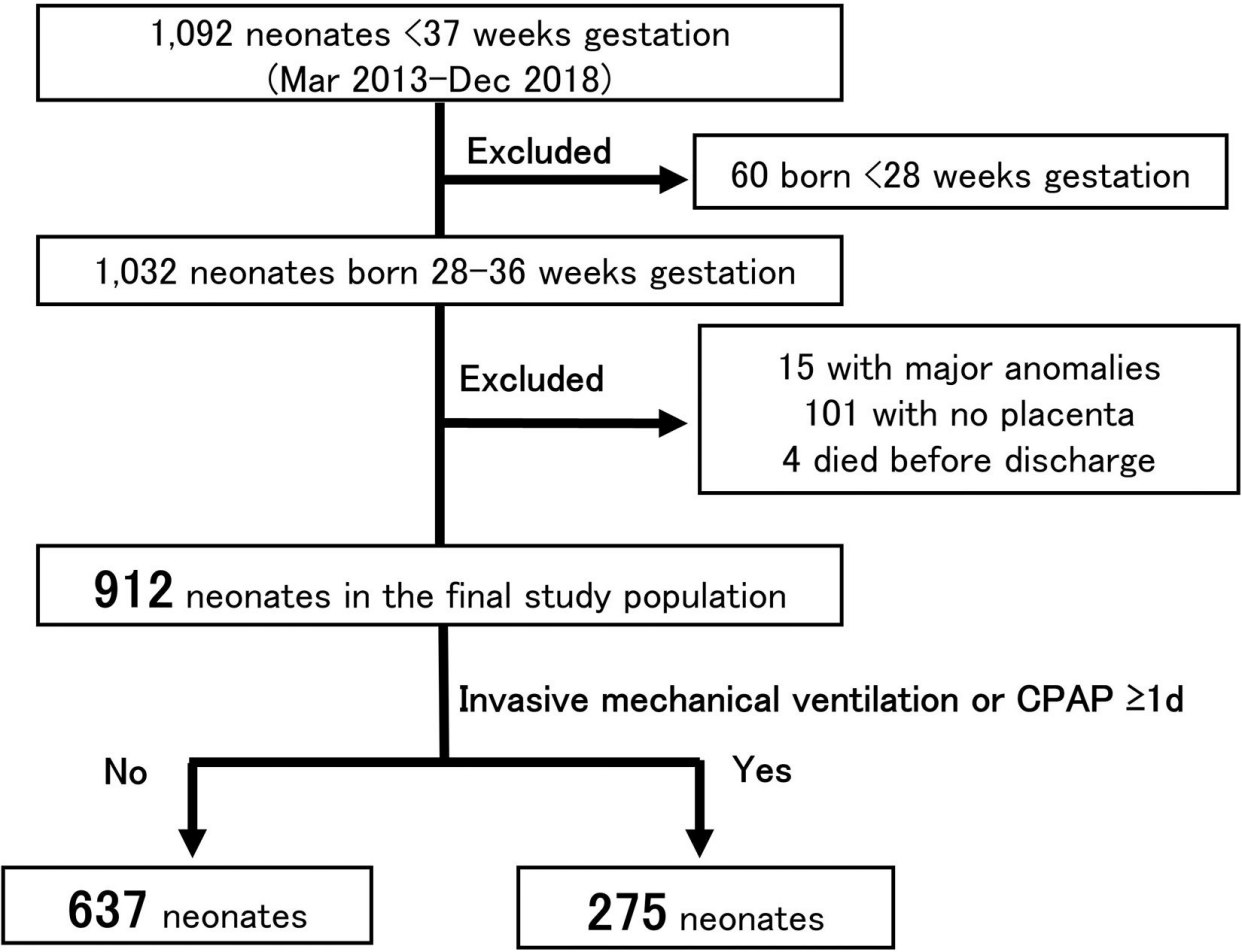
Profile of the study population.

Between March 2013 and December 2018, 1,092 preterm neonates (<37 weeks of gestation) were born at the birth center of Nagoya City University West Medical Center, a single tertiary center. We reviewed the data of 912 preterm neonates born between 28 and 36 weeks of gestation after excluding those born before 28 weeks (*n* = 60). At this center, histopathological placental examination is routinely performed for all preterm births. The following neonates were excluded: 101 without placental samples, 15 with major congenital anomalies, four who died before discharge, one with anencephaly, one with intrauterine *Listeria* infection, one with fetomaternal transfusion, and one with hyperkalemia (Figure 1).

### Clinical variables

Clinical records were reviewed for background characteristics, treatments, and clinical findings. Maternal variables included multiple births, antenatal steroid use, parity, premature rupture of the membranes, placenta previa, hypertensive disorders of pregnancy, gestational diabetes mellitus, gestational age at delivery, chorioamnionitis, and funisitis. Neonatal variables included birth weight and *Z*-score, sex, Apgar scores, need for respiratory support at birth, and laboratory results from cord and venous blood obtained at admission. Meconium-stained amniotic fluid, defined as the presence of meconium in the amniotic fluid, was visually assessed either at the time of membrane rupture or during labor (17). All placentas and umbilical cords were examined by an experienced pathologist. Chorioamnionitis was defined as Blanc's classification stage II or greater, indicating neutrophil infiltration of the chorionic plate (18), and funisitis as neutrophil infiltration within the walls of umbilical vessels or Wharton's jelly (19).

### Laboratory studies

At this center, serum C-reactive protein (CRP), α_1_-acid glycoprotein (α_1_‐AG), and haptoglobin are routinely measured using venous blood obtained at admission; cord blood is used for neonates not admitted to the Neonatal Intensive Care Unit (NICU). All assays were performed using a turbidimetric immunoassay (Quick Turbo, Shino-Test, Tokyo, Japan). Positivity was defined as CRP ≥0.3 mg/dL, α_1_-AG ≥20 mg/dL, and haptoglobin ≥13 mg/dL. An Acute-Phase Inflammatory Reaction Score was assigned: 0 (no positive biomarker), 1 (one positive), 2 (two positive), and 3 (all three biomarkers positive) (Online Supplementary Table) (20–22).

### Statistical analysis

Values are presented as mean ± standard deviation (SD), number (%), or median (interquartile range) unless otherwise specified. The primary outcome was short-term respiratory morbidity requiring invasive mechanical ventilation or continuous positive airway pressure for ≥24 h, thereby excluding respiratory support provided solely for immediate postnatal resuscitation or stabilization. Univariable logistic regression was used to assess crude associations between clinical variables and respiratory morbidity. Results from the logistic regression analysis are presented as odds ratios (ORs) with 95% confidence intervals (CIs). No adjustments were made for multiple comparisons in univariable analysis because of its exploratory nature. To assess the influence of gestational age, clinical variables, and their interactions on the incidence of respiratory morbidity, generalized linear models were applied. Interaction terms were tested for variables that showed significant associations in the univariable analysis, inflammation-related variables, and those previously reported to influence respiratory outcomes. Gestational age was mean-centered before analysis. *Post-hoc* simple slope analyses were performed for predetermined independent variables (chorioamnionitis and funisitis) and for those demonstrating significant interactions with gestational age in the univariable analysis. The simple slope analysis estimated the slope of regression lines for the relationships between the independent variables and respiratory morbidity at two representative gestational ages: “low” (1 SD below the mean) and “high” (1 SD above the mean). Statistical analyses were conducted using SPSS Statistics version 28 (IBM Corp, Armonk, NY, USA). A *p*-value <0.05 was considered statistically significant.

## Results

The final study cohort comprised 912 neonates with a mean gestational age of 34.9 ± 1.6 weeks and mean birth weight of 2,158 ± 405 g. Multiple births occurred in 279 cases (30.6%), and hypertensive disorders of pregnancy in 125 (13.7%). Antenatal steroids were administered in 149 cases (16.3%). Premature rupture of membranes lasting >24 h occurred in 119 cases (13.1%). Cesarean delivery was performed in 575 neonates (63.0%). Overall, 275 neonates (30.2%) required respiratory support. Chorioamnionitis and funisitis were diagnosed in 106 (11.6%) and 127 (13.9%) neonates, respectively.

### Influence of clinical variables on respiratory morbidity

Among maternal variables, cesarean delivery (*p* = .029), multiple birth (*p* = .027), chorioamnionitis (*p* < .001), funisitis (*p* = .045), and antenatal steroid use (*p* < .001) were associated with increased incidence of respiratory morbidity. Among postnatal clinical variables, lower gestational age (*p* < .001), lower birth weight (*p* < .001), male sex (*p* = .045), lower 1-minute and 5-minute Apgar scores (both *p* < .001), lower cord blood pH (*p* < .001), elevated CRP (*p* = .001), elevated α_1_-AG (*p* = .037), elevated haptoglobin (*p* = .010), and an acute-phase inflammatory reaction score of 3 (*p* = .023, compared with score 0) were associated with the incidence of respiratory morbidity (Table 1).

**Table 1:** Univariate analysis of independent variables for respiratory morbidities.

Variable	Respiratory support		Odds ratio			*p*-value
	No	Yes	Mean	95% CI		
	*n* = 637	*n* = 275		Lower	Upper	
Gestational age (weeks)	35.7 ± 1.2	33.2 ± 2.4	0.5	0.4	0.5	<.001
Birth-weight (g)^a^	2291 ± 443	1872 ± 527	0.8	0.8	0.9	<.001
Birth-weight *Z*-score	−0.36 ± 1.16	−0.4 ± 1.21	1.0	0.9	1.1	.735
Male sex	331 (52)	123 (44.7)	0.7	0.6	1.0	.045
Cesarean delivery						
All	387 (60.8)	188 (68.4)	1.4	1.0	1.9	.029
With labor	78 (12.2)	93 (33.8)	3.6	2.5	5.1	<.001
PROM >24 h	76 (11.9)	43 (15.6)	1.4	0.9	2.0	.128
Placenta previa	42 (6.6)	17 (6.2)	0.9	0.5	1.7	.817
Multiple birth	209 (32.8)	70 (25.5)	0.7	0.5	1.0	.027
HDP	79 (12.4)	46 (16.7)	1.4	1.0	2.1	.082
GDM	26 (4.1)	16 (5.8)	1.5	0.8	2.8	.253
Antenatal steroid	59 (9.3)	90 (32.7)	4.8	3.3	6.9	<.001
1-minute Apgar score	8 (8-8)	7 (5-8)	0.5	0.4	0.6	<.001
5-minute Apgar score	9 (8-9)	8 (7-8)	0.2	0.2	0.3	<.001
Meconium-stained amniotic fluid	25 (3.9)	18 (6.6)	1.7	0.9	3.2	.091
Cord blood pH^b^	7.27 ± 0.07	7.24 ± 0.11	0.6	0.5	0.8	<.001
Days of mechanical ventilation	0 (0-0)	3 (2-6)	75.1	30.6	184.3	<.001
Invasive ventilation	33 (5.2)	251 (91.2)	191.4	110.9	330.5	<.001
Invasive ventilation ≥3 days	0 (0)	146 (53)	119.0	51.5	275.2	<.001
Chorioamnionitis	57 (9)	49 (17.5)	2.2	1.5	3.3	<.001
Funisitis	79 (12.4)	48 (17.5)	1.5	1.0	2.2	.045
Inflammatory biomarkers from umbilical cord blood and venous sample at admission						
CRP ≥ 0.3 mg/dL	5 (0.8)	9 (3.3)	4.2	1.4	12.7	.001
α_1_‐AG ≥ 20 mg/dL	39 (6.2)	28 (10.2)	1.7	1.0	2.8	.037
Haptoglobin ≥ 13 mg/dL	12 (1.9)	13 (4.7)	2.6	1.1	5.7	.01
Acute-phase inflammatory reaction score from umbilical cord blood and venous sample at admission^c^						
0	590 (93.8)	246 (89.5)	Reference			
1	26 (4.1)	15 (5.5)	1.4	0.7	2.7	.329
2	9 (1.4)	7 (2.5)	1.9	0.7	5.1	.221
3	4 (0.6)	7 (2.5)	4.2	1.2	14.5	.023

Values were expressed as the number (%), mean ± standard deviation, or median (quartile ranges). α_1_‐AG, α_1_-acid glycoprotein; CI, confidence interval; CRP, c-reactive protein; GDM, gestational diabetes mellitus; HDP, hypertensive disorder of pregnancy; PROM, premature rupture of the membrane.

Odds ratios were calculated per 100 g^a^ and 0.10 pH^b^ changes.

c Acute-phase inflammatory reaction scores of 0–3 were assigned according to the elevation of CRP, α_1_-AG, and haptoglobin (see Online Supplementary Material 1).

### Gestational age–specific influence of clinical variables on respiratory morbidity

Interactions with gestational age were identified for birth-weight *Z*-score (OR 0.9, 95% CI 0.8–1.0, *p* = .002), cesarean delivery (OR 1.6, 95% CI 1.2–2.1, *p* = .001), and funisitis (OR 0.6, 95% CI 0.4–0.9, *p* = .019) but not for chorioamnionitis (OR 0.7, 95% CI 0.5–1.0, *p* = .055) (Table 2).

**Table 2:** Influence of gestational age, clinical variables, and their interaction on respiratory morbidities.

Variable	Odds ratio			*p*-value
	Mean	95% CI		
		Lower	Upper	
Birth weight (g)^a^	1.0	1.0	1.1	.276
Gestational age	0.6	0.4	0.9	.008
Gestational age × birth weight (g)^a^	1.0	1.0	1.0	.149
Birth-weight *Z*-score	1.1	0.9	1.2	.491
Gestational age	0.4	0.4	0.5	<.001
Gestational age × birth weight *Z*-score	0.9	0.8	1.0	.002
Male sex	0.9	0.6	1.2	.402
Gestational age	0.5	0.5	0.6	<.001
Gestational age × male sex	0.9	0.7	1.1	.279
Cesarean delivery	1.4	0.9	2.0	.102
Gestational age	0.3	0.3	0.4	<.001
Gestational age × cesarean delivery	1.6	1.2	2.1	.001
Multiple birth	0.9	0.6	1.3	.688
Gestational age	0.5	0.4	0.5	<.001
Gestational age × multiple birth	1.2	0.9	1.5	.175
Antenatal steroid	1.0	0.5	1.9	.957
Gestational age	0.4	0.4	0.6	<.001
Gestational age × antenatal steroid	1.1	0.8	1.4	.608
1-minute Apgar score	0.6	0.5	0.7	<.001
Gestational age	0.4	0.2	0.8	.012
Gestational age × 1-minute Apgar score	1.0	0.9	1.1	.631
5-minute Apgar score	0.3	0.2	0.4	<.001
Gestational age	0.7	0.2	2.5	.591
Gestational age × 5-minute Apgar score	1.0	0.8	1.1	.556
Cord blood pH^b^	0.5	0.4	0.7	<.001
Gestational age	0.5	0.4	0.5	<.001
Gestational age × cord blood pH^b^	1.0	0.9	1.2	.745
Chorioamnionitis	1.2	0.6	2.1	.619
Gestational age	0.5	0.4	0.6	<.001
Gestational age × chorioamnionitis	0.7	0.5	1.0	.055
Funisitis	1.0	0.6	1.8	.974
Gestational age	0.5	0.5	0.6	<.001
Gestational age × funisitis	0.6	0.4	0.9	.019

Odds ratios were calculated per 100 g^a^ and 0.10 pH^b^ changes. Gestational age and 0.10 pH were analyzed using mean-centered values.

Simple slope analysis was performed to estimate the slope of regression between independent variables and the incidence of respiratory morbidity at low (33.4 weeks) and high (36.5 weeks) gestational ages. A higher birth-weight *Z*-score (OR 1.4, 95% CI 1.1–1.7, *p* = .002), chorioamnionitis (OR 2.5, 95% CI 1.1–5.9, *p* = .034), and funisitis (OR 2.4, 95% CI 1.0–5.5, *p* = .039) were associated with increased incidence of respiratory morbidity at low gestational ages only. In contrast, cesarean delivery was associated with increased incidence of respiratory morbidity at high gestational ages (OR 3.5, 95% CI 1.7–7.1, *p* < .001), but not at low gestational ages (Table 3).

**Table 3:** Independent variables for respiratory morbidities between low and high gestational age.

Variable	Low gestational age			*p*-value	High gestational age			*p*-value
	Odds ratio				Odds ratio			
	Mean	95% CI			Mean	95% CI		
		Lower	Upper			Lower	Upper	
Birth-weight *Z*-score	1.4	1.1	1.7	.002	0.8	0.6	1.0	.084
Cesarean delivery	0.5	0.3	1.0	.052	3.5	1.7	7.1	<.001
Chorioamnionitis	2.5	1.1	5.9	.034	0.5	0.2	1.7	.280
Funisitis	2.4	1.0	5.5	.039	0.4	0.1	1.1	.089

Simple slopes were calculated for 1 standard deviation below (low, 33.4 weeks) and above (high, 36.5 weeks) the mean gestational age.

## Discussion

In this study, we demonstrated that antenatal inflammation, represented by chorioamnionitis and funisitis, was associated with adverse short-term respiratory outcomes only at low gestational ages. A higher birth-weight *Z*-score showed a similar interaction pattern, being associated with respiratory morbidity only at low gestational ages. Meanwhile, caesarean delivery was associated with respiratory morbidity only in high gestational ages. These findings suggest that intrauterine inflammation and excessive fetal growth disproportionately affect more immature neonates, whereas cesarean delivery poses a greater risk among more mature preterm neonates. Gestational age should be carefully considered when determining the optimal mode and timing of delivery.

### Intrauterine inflammation and gestational age

The impact of infection and inflammation on the lungs has been extensively investigated in extremely preterm neonates because of their high incidence of long-term respiratory morbidity, most notably bronchopulmonary dysplasia, and its lifelong consequences (3, 4). Intrauterine inflammation appears to exert both beneficial and deleterious effects on the developing lung. On one hand, inflammatory exposure may induce pulmonary injury, alter vascular permeability, and impair respiratory adaptation after birth. Recent studies have also reported reduced breathing effort at birth among premature infants exposed to chorioamnionitis or funisitis (23). On the other hand, intrauterine inflammation may accelerate pulmonary maturation and surfactant synthesis, leading to a reduced incidence of acute respiratory disorders in some observational studies (9, 10, 12).

The clinical implications of inflammation may extend beyond extremely preterm neonates. Meconium aspiration syndrome, traditionally regarded as a consequence of airway obstruction and lung inflammation triggered by fetal hypoxia-ischemia and subsequent meconium passage, is now thought to involve systemic inflammation. Lung injury may precede aspiration of contaminated amniotic fluid, with fetal exposure to pro-inflammatory cytokines possibly inducing lung injury and stimulating bowel peristalsis, leading to meconium passage (17, 20, 21). Our group previously reported that inflammation-related respiratory morbidities are common among neonates born at 32–34 weeks of gestation (22). Nevertheless, data on the impact of inflammation on lung development and respiratory outcomes among preterm neonates born after 28 weeks remains limited.

In the present study, we found that intrauterine inflammation adversely affected short-term respiratory outcomes only among those with relatively lower gestational ages. Compared to many previous studies reporting a beneficial impact of intrauterine inflammation on short-term neonatal respiratory function (9, 10, 12), our study included relatively more mature neonates. Previous studies suggested that background variables such as antenatal glucocorticoid administration influence the impact of inflammation on the incidence of short-term respiratory morbidities. Since the 2000s, antenatal steroid administration has become widely adopted, particularly in Western countries, with reported administration rates of 80%–90% among neonates born before 34 weeks of gestation (24). Although intrauterine inflammation reportedly improves short-term respiratory outcomes in preterm neonates, such benefits may reflect the effects of antenatal steroid use rather than the inflammation itself. Because extremely preterm neonates are both more likely to receive antenatal steroids and more frequently born due to intrauterine inflammation, separating the independent effects of inflammation remains challenging. Given the complex interplay among these various factors, future studies with larger sample sizes may be necessary to clarify the independent effect of inflammation on respiratory morbidity.

### Large birth-weight *Z*-score and gestational age

Intrauterine growth restriction is a known risk factor for chronic lung disease. However, its effects on short-term respiratory outcomes may differ, as accumulating evidence suggests an inverse relationship between organ maturation and fetal growth (25). Antenatal stress, such as that resulting from prolonged placental dysfunction, may stimulate endogenous cortisol production (14), accelerating lung maturation and potentially reducing the incidence of acute respiratory complications (26). Conversely, large-for-gestational-age fetuses, who experience less intrauterine stress and greater nutrient supply, exhibit delayed lung development in preclinical models (27). Furthermore, even in the absence of maternal diabetes, large-for-gestational-age neonates have been reported to exhibit higher cord blood insulin and C-peptide concentrations than appropriate-for-gestational-age neonates (28, 29), suggesting relative fetal hyperinsulinemia, which may inhibit surfactant production and delay lung maturation. In addition, delayed cardiopulmonary adaptation after birth has been reported in large-for-gestational-age neonates born to both diabetic and nondiabetic mothers (25), which may increase susceptibility to respiratory morbidity during the early neonatal period. Consistent with our previous findings in neonates born at 32–34 weeks (22), the present study demonstrated that greater fetal growth was associated with an increased risk of short-term respiratory morbidity, particularly among less mature preterm neonates. Although “greater fetal growth” is often perceived as reassuring and favorable, our findings indicate that excessive fetal growth may delay lung maturation and contribute to short-term respiratory morbidity.

### Mode of delivery and gestational age

Cesarean sections performed for indications other than emergencies‒such as breech presentation, multiple gestation, or prior cesarean delivery‒are becoming increasingly common (30). The World Health Organization has cautioned that the rising use of labor induction and cesarean delivery has contributed to the global increase in moderate-to-late preterm births (31). Among near-term and term neonates, cesarean delivery without labor is associated with a higher risk of respiratory disorders, potentially owing to reduced catecholamine release, absence of thoracic compression, and delayed clearance of lung fluid (32). Consequently, cesarean deliveries performed for non-medical reasons may impose significant health burdens and increase healthcare resource use. Despite the importance of weighing its benefit against potential adverse outcomes, little is known about the gestational age–specific effects of cesarean delivery on neonatal respiratory transition.

We found that cesarean delivery was associated with an increased incidence of short-term respiratory morbidity only in neonates with relatively higher gestational ages. Performing cesarean delivery in relatively more preterm neonates up to 34 weeks of gestation may not confer additional risk of short-term respiratory morbidity. Conversely, cesarean delivery increased the odds of developing short-term respiratory morbidity by 3.5-fold in neonates born at approximately 35‒36 weeks of gestation. Considering that the effect of cesarean delivery on short-term respiratory morbidity was among the most prominent effects observed in this study, further dissemination of these findings may be warranted to minimize non-medical cesarean deliveries during the late-preterm period.

### Strengths and limitations

Using a relatively large study population, we could assess the gestational age–specific roles of inflammation and other important variables in the development of short-term respiratory morbidities. The effect of inflammation was evaluated using histological examination of the placenta. Because the study population included neonates across a wide range of gestational ages, we could examine gestational age–dependent associations between antenatal inflammation, additional clinical factors, and respiratory outcomes. Nevertheless, some limitations must be noted. Neonates born before 28 weeks of gestation were not included because their short-term respiratory morbidity is primarily determined by lung immaturity; indeed, most extremely preterm neonates require invasive respiratory support. We evaluated only short-term respiratory morbidities; therefore, the relationship among antenatal inflammation, other clinical background variables, long-term respiratory outcomes, neurodevelopmental outcomes, and economic impact remains unknown. Furthermore, although we assessed acute-phase reactants such as CRP, α_1_-acid glycoprotein, and haptoglobin to capture antenatal inflammation across different pathways, we were unable to evaluate pro-inflammatory cytokine responses. Future studies may explore cytokine profiles in amniotic fluid and other biological samples to better characterize gestational age–specific inflammatory responses and subsequent incidence of respiratory morbidities. Finally, although major clinical confounders were included in the multivariable models, residual confounding resulting from the coexistence of multiple perinatal conditions may still be present. Therefore, the observed associations should be interpreted with caution. More detailed obstetric and perinatal information may help further clarify these findings.

## Conclusion

Intrauterine inflammation was associated with adverse short-term respiratory morbidities only in neonates born at relatively low gestational ages, a pattern that is consistent with observations in the current perinatal care environment, where antenatal glucocorticoids are widely used. This finding also aligns with earlier reports suggesting that intrauterine inflammation exerts greater deleterious impact on relatively immature lungs. The birth-weight *Z*-score was inversely associated with respiratory morbidity among neonates with relatively lower gestational ages, indicating that large-for-gestational-age may not necessarily confer a favorable respiratory transition shortly after birth. Our study also confirmed that the risk of respiratory morbidity associated with cesarean delivery was evident only among neonates of relatively higher gestational age, supporting strategies aimed at avoiding non-medically indicated cesarean deliveries while not hesitating to use this delivery mode for medical indications in more premature neonates. Further research is needed to confirm the gestational age–specific roles of inflammation and other independent variables in the development of short- and long-term respiratory morbidities among neonates with diverse gestational stages and clinical backgrounds. Nevertheless, because only gestational age was adjusted for, residual confounding by other factors cannot be excluded, and these findings should be interpreted with caution.

## Data Availability

The datasets presented in this article are not readily available because patient privacy and ethical restrictions apply. Requests to access the datasets should be directed to kyoyo@med.nagoya-cu.ac.jp.

## References

[1] Serenius F Ewald U Farooqi A Fellman V Hafstrom M Hellgren K. Neurodevelopmental outcomes among extremely preterm infants 6.5 years after active perinatal care in Sweden. JAMA Pediatr. (2016) 170:954–63. 10.1001/jamapediatrics.2016.1210 27479919

[2] Gantert M Been JV Gavilanes AW Garnier Y Zimmermann LJ Kramer BW. Chorioamnionitis: a multiorgan disease of the fetus? J Perinatol. (2010) 30(Suppl):S21–30. 10.1038/jp.2010.96 20877404

[3] Nakashima T Inoue H Sakemi Y Ochiai M Yamashita H Ohga S. Trends in bronchopulmonary dysplasia among extremely preterm infants in Japan, 2003–2016. J Pediatr. (2021) 230:119–25.e7. 10.1016/j.jpeds.2020.11.041 33246013

[4] Geetha O Rajadurai VS Anand AJ Dela Puerta R Huey Quek B Khoo PC. New BPD-prevalence and risk factors for bronchopulmonary dysplasia/mortality in extremely low gestational age infants ≤28 weeks. J Perinatol. (2021) 41:1943–50. 10.1038/s41372-021-01095-6 34031514 PMC8280382

[5] Backstrom E Hogmalm A Lappalainen U Bry K. Developmental stage is a major determinant of lung injury in a murine model of bronchopulmonary dysplasia. Pediatr Res. (2011) 69:312–8. 10.1203/PDR.0b013e31820bcb2a 21178818

[6] Burri PH. Fetal and postnatal development of the lung. Annu Rev Physiol. (1984) 46:617–28. 10.1146/annurev.ph.46.030184.003153 6370120

[7] Kallapur SG Willet KE Jobe AH Ikegami M Bachurski CJ. Intra-amniotic endotoxin: chorioamnionitis precedes lung maturation in preterm lambs. Am J Physiol Lung Cell Mol Physiol. (2001) 280:L527–36. 10.1152/ajplung.2001.280.3.L527 11159037

[8] Kramer BW Kramer S Ikegami M Jobe AH. Injury, inflammation, and remodeling in fetal sheep lung after intra-amniotic endotoxin. Am J Physiol Lung Cell Mol Physiol. (2002) 283:L452–9. 10.1152/ajplung.00407.2001 12114208

[9] Shimoya K Taniguchi T Matsuzaki N Moriyama A Murata Y Kitajima H. Chorioamnionitis decreased incidence of respiratory distress syndrome by elevating fetal interleukin-6 serum concentration. Hum Reprod. (2000) 15:2234–40. 10.1093/humrep/15.10.2234 11006206

[10] Been JV Rours IG Kornelisse RF Lima Passos V Kramer BW Schneider TA. Histologic chorioamnionitis, fetal involvement, and antenatal steroids: effects on neonatal outcome in preterm infants. Am J Obstet Gynecol. (2009) 201:587.e1–8. 10.1016/j.ajog.2009.06.025 19729143

[11] Lahra MM Beeby PJ Jeffery HE. Maternal versus fetal inflammation and respiratory distress syndrome: a 10-year hospital cohort study. Arch Dis Child Fetal Neonatal Ed. (2009) 94:F13–6. 10.1136/adc.2007.135889 18463119

[12] Park CW Park JS Jun JK Yoon BH. Mild to moderate, but not minimal or severe, acute histologic chorioamnionitis or intra-amniotic inflammation is associated with a decrease in respiratory distress syndrome of preterm newborns without fetal growth restriction. Neonatology. (2015) 108:115–23. 10.1159/000430766 26087777

[13] Metcalfe A Lisonkova S Sabr Y Stritzke A Joseph KS. Neonatal respiratory morbidity following exposure to chorioamnionitis. BMC Pediatr. (2017) 17:128. 10.1186/s12887-017-0878-9 28514958 PMC5436447

[14] Iwata S Kinoshita M Okamura H Tsuda K Saikusa M Harada E. Intrauterine growth and the maturation process of adrenal function. PeerJ. (2019) 7:e6368. 10.7717/peerj.6368 30746307 PMC6368969

[15] Hansen AK Wisborg K Uldbjerg N Henriksen TB. Risk of respiratory morbidity in term infants delivered by elective caesarean section: cohort study. Br Med J. (2008) 336:85–7. 10.1136/bmj.39405.539282.BE 18077440 PMC2190264

[16] Ohuma EO Moller AB Bradley E Chakwera S Hussain-Alkhateeb L Lewin A. National, regional, and global estimates of preterm birth in 2020, with trends from 2010: a systematic analysis. Lancet. (2023) 402:1261–71. 10.1016/S0140-6736(23)00878-4 37805217

[17] Lee J Romero R Lee KA Kim EN Korzeniewski SJ Chaemsaithong P. Meconium aspiration syndrome: a role for fetal systemic inflammation. Am J Obstet Gynecol. (2016) 214:366.e1–9. 10.1016/j.ajog.2015.10.009 26484777 PMC5625352

[18] Blanc WA. Pathology of the placenta and cord in ascending and in haematogenous infection. Ciba Found Symp. (1979) 77:17–38. 10.1002/9780470720608.ch3 261758

[19] Redline RW Faye-Petersen O Heller D Qureshi F Savell V Vogler C. Amniotic infection syndrome: nosology and reproducibility of placental reaction patterns. Pediatr Dev Pathol. (2003) 6:435–48. 10.1007/s10024-003-7070-y 14708737

[20] Yokoi K Iwata O Kobayashi S Muramatsu K Goto H. Influence of foetal inflammation on the development of meconium aspiration syndrome in term neonates with meconium-stained amniotic fluid. PeerJ. (2019) 7:e7049. 10.7717/peerj.7049 31183262 PMC6546081

[21] Yokoi K Iwata O Kobayashi S Kobayashi M Saitoh S Goto H. Evidence of both foetal inflammation and hypoxia-ischaemia is associated with meconium aspiration syndrome. Sci Rep. (2021) 11:16799. 10.1038/s41598-021-96275-x 34408219 PMC8373916

[22] Yokoi K Iwata O Kobayashi S Jinnou H Kato S Saitoh S. Intrauterine inflammation, excessive fetal growth and respiratory morbidities in moderate-to-late preterm neonates. Neonatology. (2023) 121:1–8. 10.1159/000534163 38104557 PMC10994573

[23] Panneflek TJR Dekker J Kuypers K Van Der Meeren LE Polglase GR Hooper SB. The effect of histological and subclinical chorioamnionitis and funisitis on breathing effort in premature infants at birth: a retrospective cohort study. Eur J Pediatr. (2024) 183:5497–507. 10.1007/s00431-024-05815-w 39453483 PMC11527944

[24] Ehret DEY Edwards EM Greenberg LT Bernstein IM Buzas JS Soll RF. Association of antenatal steroid exposure with survival among infants receiving postnatal life support at 22 to 25 Weeks’ gestation. JAMA Netw Open. (2018) 1:e183235. 10.1001/jamanetworkopen.2018.3235 30646235 PMC6324435

[25] Vela-Huerta M Aguilera-Lopez A Alarcon-Santos S Amador N Aldana-Valenzuela C Heredia A. Cardiopulmonary adaptation in large for gestational age infants of diabetic and nondiabetic mothers. Acta Paediatr. (2007) 96:1303–7. 10.1111/j.1651-2227.2007.00414.x 17718783

[26] Lal MK Manktelow BN Draper ES Field DJ. Chronic lung disease of prematurity and intrauterine growth retardation: a population-based study. Pediatrics. (2003) 111:483–7. 10.1542/peds.111.3.483 12612225

[27] Heyob KM Mieth S Sugar SS Graf AE Lallier SW Britt RD Jr Maternal high-fat diet alters lung development and function in the offspring. Am J Physiol Lung Cell Mol Physiol. (2019) 317:L167–74. 10.1152/ajplung.00331.2018 31042079 PMC6734382

[28] Akinbi HT Gerdes JS. Macrosomic infants of nondiabetic mothers and elevated C-peptide levels in cord blood. J Pediatr. (1995) 127:481–4. 10.1016/S0022-3476(95)70087-0 7658284

[29] Hou RL Jin WY Chen XY Jin Y Wang XM Shao J. Cord blood C-peptide, insulin, HbA1c, and lipids levels in small- and large-for-gestational-age newborns. Med Sci Monit. (2014) 20:2097–105. 10.12659/MSM.890929 25357084 PMC4226317

[30] Vogel JP Betran AP Vindevoghel N Souza JP Torloni MR Zhang J. Use of the Robson classification to assess caesarean section trends in 21 countries: a secondary analysis of two WHO multicountry surveys. Lancet Glob Health. (2015) 3:e260–70. 10.1016/S2214-109X(15)70094-X 25866355

[31] UNICEF, World Health Organization, PMNCH. Survive and Thrive: Transforming Care for Every Small and Sick Newborn. New York: UNICEF (2020).

[32] Alhassen Z Vali P Guglani L Lakshminrusimha S Ryan RM. Recent advances in pathophysiology and management of transient tachypnea of newborn. J Perinatol. (2021) 41:6–16. 10.1038/s41372-020-0757-3 32753712 PMC11867197

