# Supplementary material for: Gestational age–dependent effects of intrauterine inflammation, delivery mode, and fetal growth on respiratory morbidity in preterm neonates
Source: Front Pediatr. 2026 Aug 4;14:1823540. doi: 10.3389/fped.2026.1823540 (PMC13481856; doi:10.3389/fped.2026.1823540)
Supplement: Supplementary file 1 [file Datasheet1.pdf]

Online Supplementary Material

Online Supplementary Table: Definition of Acute-phase inflammatory response score

Score	Number of positive biomarkers	Criteria for positivity
0	None	–
1	One	CRP ≥ 0.3 mg/dL or α_1-AG ≥ 20 mg/dL or haptoglobin ≥ 13 mg/dL
2	Two	Any two positive biomarkers
3	Three	All three biomarkers positive

Abbreviations: α_1-AG, α_1-acid glycoprotein; CRP, c-reactive protein
